# Analysis of factors influencing pregnancy and its outcomes in women undergoing in vitro fertilization–embryo transfer/frozen embryo transfer cycles: A retrospective study

**DOI:** 10.1097/MD.0000000000039110

**Published:** 2024-08-02

**Authors:** Hao Zhang, Yuan yuan Zhang, Yaping Cheng, Hua Yan, Xi Zheng

**Affiliations:** a Reproductive Medicine Center, Yanbian University Hospital, Yanji, Jilin Province, China.

**Keywords:** elective double embryo transfer, elective single-embryo transfer, in vitro fertilization–embryo transfer/frozen embryo transfer, multiple pregnancy, pregnancy rate

## Abstract

The relationship between clinical outcomes and various factors influencing pregnancy was analyzed to provide reference data for patients and clinicians when selecting embryo transfer protocols. This was a retrospective study of 1309 transfer cycles between June 1, 2018, and May 1, 2023, in the Reproductive Medicine Center. Univariate analysis was performed on various factors that may have affected pregnancy outcomes, and further regression analysis was performed on those factors found by univariate analysis to correlate positively with clinical pregnancy outcomes. Finally, the embryo transfer schemes were compared based on the analysis results. The results showed that the stage of embryonic development significantly affected pregnancy outcomes after transplantation (*P* < .01, 95% confidence interval: 2.554 [1.958–3.332]). There was no significant difference in the pregnancy rate between 1 high-quality blastocyst transfer and 2 cleavage-stage embryos or blastocyst transfer (64.22% vs 70.11%, *P* = .439); however, the rate of multiple pregnancies after 1 high-quality blastocyst transfer was close to the rate of natural conception. These data show that the transfer of single high-quality blastocysts can significantly reduce the multiple pregnancy rate while ensuring an ideal pregnancy rate, which can be used as a reference for planning the first transplantation in patients with good prognoses.

## 1. Introduction

Since the birth of the first test-tube baby in 1978, in vitro fertilization (IVF)/embryo transfer has helped many infertile couples to give birth to their offspring. The success rate of IVF-related clinical pregnancy has improved significantly as a result of technological developments and progress.^[1]^ When infertile couples insist on attempting IVF, the cumulative pregnancy rate may be equivalent to the rate of pregnancy after natural conception.^[2]^ However, the rate of multiple pregnancies after assisted reproductive technology is significantly higher than that of naturally conceived pregnancies, which is of concern because multiple pregnancies carry a greater risk of adverse perinatal outcomes, such as premature delivery and low birth weight.^[3]^ This issue requires attention from the assisted reproductive community. Continuous improvement in pregnancy and implantation rates is undeniably the core goal of reproductive centers. Important factors affecting the success rate include the patient’s basic characteristics, number of oocytes obtained, number of available embryos, quality of embryos, and number of embryos transferred.^[4]^ In addition, medication regimens can influence pregnancy outcomes.^[5]^ The primary goals of clinicians administering IVF are to accurately assess fertility and formulate the best plan to obtain sufficient numbers and quality gametes. In contrast, the most important task for IVF laboratory staff is to obtain a sufficient number and quality of available embryos after IVF and in vitro culture. However, it is insufficient for laboratory staff and clinicians to meet their respective responsibilities. Both groups need to collaborate to devise an optimal protocol for embryo transfer. The main purpose of these protocols is to maximize the probability of clinical pregnancy while minimizing adverse perinatal outcomes such as multiple pregnancies. This study aimed to achieve better pregnancy outcomes than the highest probability of pregnancy. An increasing number of recent studies have focused on elective single-embryo transfers (e-SETs). Compared with traditional transplantation methods, e-SET significantly reduces the rate of multiple pregnancies while ensuring that the pregnancy rate is not compromised.^[6]^ The rate of multiple pregnancies after e-SET was similar to that after natural conception.^[7]^ and a significantly lower risk of preterm birth and low birth weight.^[8]^ In addition, some research has suggested that recommending e-SET to patients with better prognoses results in better pregnancy outcomes.^[9]^ However, this possibility needs to be verified in large, multicenter studies.

Clinical pregnancy rate is a crucial metric for assessing the excellence of reproductive centers. Improving the clinical pregnancy rate while reducing the incidence of perinatal complications is a long-term goal of clinical and laboratory work. Although there are many factors affecting the clinical pregnancy rate of the transfer cycle,^[10]^ the most significant factors are considered first when selecting the embryo transfer scheme, and it is worth considering developing personalized embryo transfer.^[11]^ Therefore, a comprehensive analysis based on previous transfer cycles is necessary. This study aimed to analyze the significant factors influencing the pregnancy rate in the transfer cycle and to compare the pregnancy outcomes of various embryo transfer schemes. In this process, individualized embryo culture and transfer schemes are individualized for patients with differing characteristics.

## 2. Materials and methods

### 2.1. Study cohort

The study cohort comprised 830 patients who had completed 1309 IVF cycles involving fresh or frozen embryo transfers (FETs) at the Affiliated Hospital of Yanbian University between June 1, 2018, and May 1, 2023. The cycles comprised 192 cycles using fresh embryo transfer and 1117 cycles using FET. All study patients completed the pregnancy follow-up. The patient information collected in this study included age, body mass index, infertility type, stage of transplanted embryos, number of embryos transferred, retrieved oocyte number, insemination method, endometrial thickness before transplantation, total dose of gonadotropin (Gn), total number of days of Gn, and rates of clinical and multiple pregnancies.

### 2.2. Controlled ovarian stimulation and oocyte retrieval procedure

Ovulation induction protocols were formulated by clinicians according to the patient’s characteristics and findings on the assessment of the current cycle, including a Gn-releasing hormone agonist protocol, antagonist protocol, minimal ovarian stimulation, luteal-phase ovarian stimulation protocol, and spontaneous cycles. Oocyte retrieval was performed by transvaginal ultrasound-guided follicular aspiration.

### 2.3. Fertilization, culture, and vitrification freezing

In the present study, insemination methods were IVF (short-term) and intracytoplasmic sperm injection. Embryo culture was performed using sequential culture (SAGE cryopreservation media, Trumbull, CT). Embryos were cultured for 5 to 6 days. The embryo freezing and thawing protocol included vitrification, freezing, and thawing (Kitazato Vitrification Media, Fuji, Shizuoka, Japan).

### 2.4. Scoring of embryo quality

Embryo quality was scored in accordance with the standards of the Istanbul consensus workshop on embryo assessment (2011)^[12]^ The definition of high-quality embryos was as follows: < 10% fragmentation, stage-specific cell size, and no multinucleation. High-quality blastocysts were defined as follows: inner cell mass prominent and easily discernible, with many compacted and tightly adherent cells; and trophectoderm comprising many cells forming a cohesive epithelium.

### 2.5. Embryo transfer selection

Embryos transplanted in fresh and FET cycles were selected on the basis of the patient’s informed consent. The clinician fully informed the patient of the benefits and risks of transferring 1 or 2 embryos, focusing on the pregnancy rate and risk of multiple pregnancies. If the cycle was not suitable for fresh embryo transfer or if the transfer was canceled for other reasons, the fresh cycle embryos were frozen immediately or after further culturing for FET cycle transfer.

### 2.6. Institutional review board

This study extracted and summarized data on IVF and FET cycles using an independent secure electronic database that ensured patient anonymity. No protected health information was used during the chart review. the institutional review board determined that this study did not constitute research on human subjects and did not violate any human subject’s privacy.

### 2.7. Statistical analysis

**Figure 1.**
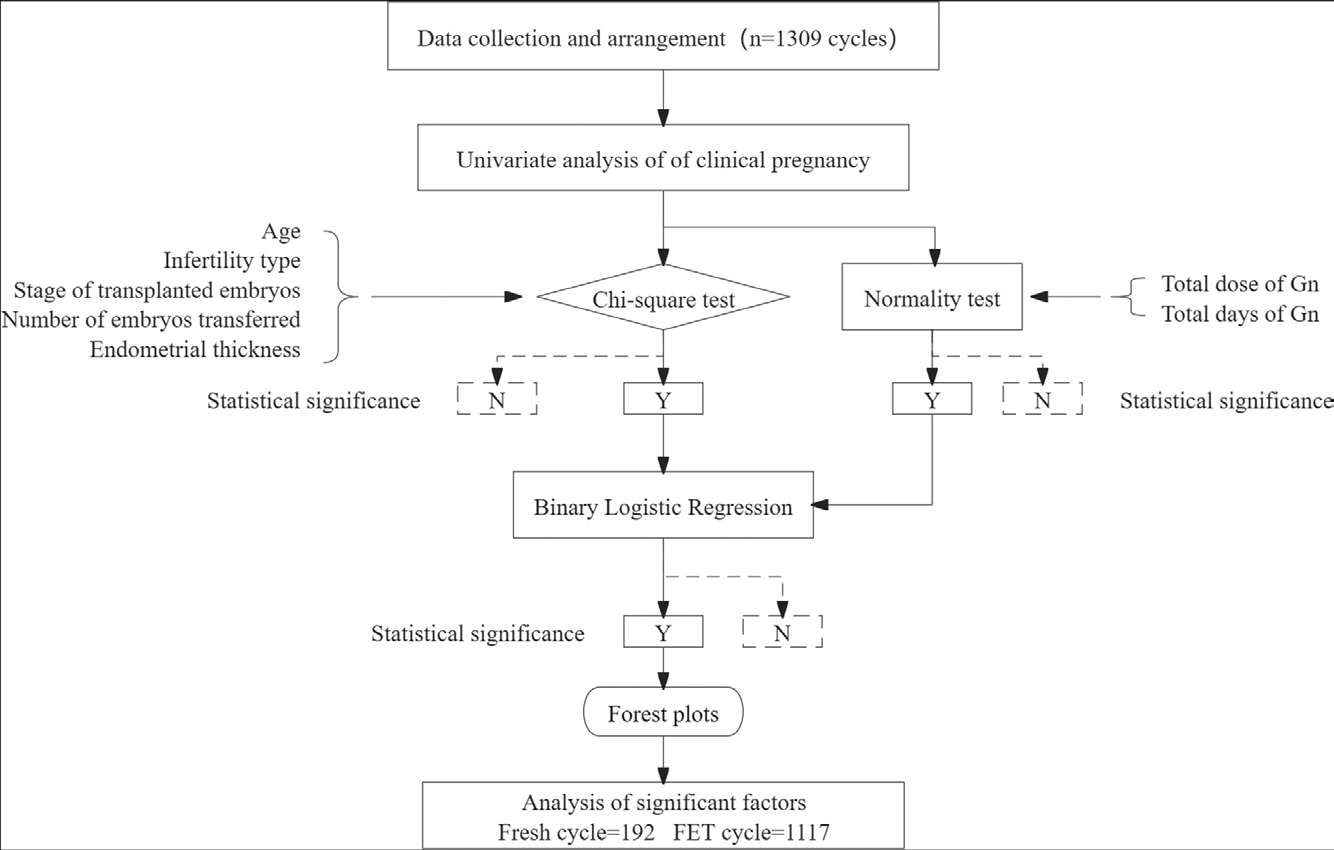
Study process diagram of correlation analysis. FET = frozen embryo transfer, Gn = gonadotropin.

SPSS 19.0 (Armonk, New York) and GraphPad Prism 8.0 (Boston, MA) were used for statistical analysis. Shapiro–Wilk tests of normality and χ^2^ tests were used for univariate analysis of pregnancy outcomes. Multivariate analysis was performed using a binary logistic regression and forest plots. Statistical significance was set at *P* < .05. The statistical procedure is illustrated in Figure 1.

## 3. Results

**Figure 2.**
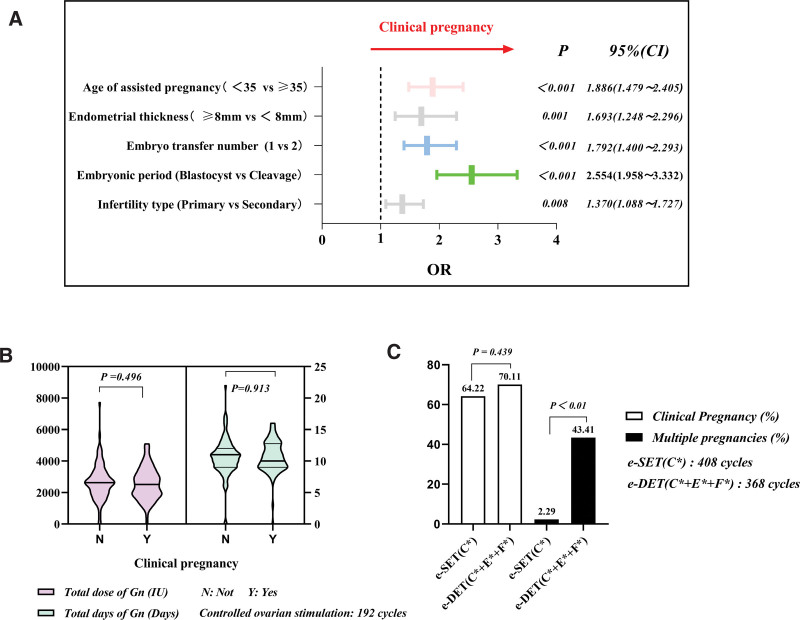
(A) Results of assessing χ^2^ test significant factors influencing pregnancy by Binary logistic regression and Forest plots. (B) Results of Gn dose/days and clinical pregnancy normality test in controlled ovarian stimulation transfer cycles. (C) Comparative analysis of transfer schemes for high pregnancy rate transfer cycles (χ^2^ test). CI = confidence interval, e-DET = elective double embryo transfer, e-SET = elective single-embryo transfer, Gn = gonadotropin, OR = odds ratio.

This study analyzed the factors influencing pregnancy and its outcomes in 1309 transplantation cycles. The results showed that pregnancy rates differed significantly between age groups (<35 and ≥35 years; *P* < .01) and between primary and secondary infertility (*P* < .01). There was a significant difference in pregnancy rates between transplanted cleavage-stage and blastocyst-stage embryos (*P* < .01). There was also a statistically significant difference in the pregnancy rate between transplantation of 1 and 2 embryos (*P* = .045) and between endometrial thicknesses ≥ 8 and <8 mm (*P* < .01). These findings suggest that the age of the woman in the assisted pregnancy cycle, primary infertility versus secondary infertility, cell-stage embryo transfer versus blastocyst transfer, number of embryos transferred, and endometrial thickness all correlate with clinical pregnancy rate. The results are presented in Table 1. There were no significant correlations between total Gn dosage and pregnancy rate (*P* = .496) or between total number of days of Gn and pregnancy rate (*P* = .913), suggesting that the total dosage of Gn and total number of days of Gn do not correlate with the clinical pregnancy rate. The results are shown in Figure 2B.

**Table 1:** Results of assessing factors influencing clinical pregnancy by the χ^2^ tests.

Cycle characteristics	Transfer cycles	Clinical pregnancy		Clinical pregnancy rate (%)	χ^2^/*Z*	*P*-value
		Y	N			
Female age (yr)					40.727	<0.01
<35	867	541	326	62.40		
≥35	442	194	248	43.89		
Body mass index (kg/m^2^)					0.246	0.620
18.5–24	120	61	59	50.83		
>24, <18.5	72	39	33	54.17		
Infertility duration (yr)					0.005	0.942
<5	733	411	322	56.07		
≥5	576	324	252	56.25		
Male age (yr)					35.617	<0.01
<35	637	405	232	63.58		
≥35	672	330	342	49.11		
Infertility type					15.412	<0.01
Primary infertility	741	451	290	60.86		
Secondary infertility	568	284	284	50.00		
Embryo development stage					44.280	<0.01
Cleavage	397	168	229	42.32		
Blastocyst	912	567	345	62.17		
Embryo transfer number					4.199	0.045
1	624	332	292	53.21		
2	685	403	282	58.83		
Endometrial thickness (mm)					17.503	<0.01
≥8	1084	637	447	58.76		
<8	225	98	127	43.56		
Retrieved oocyte number					0.265	0.607
<10	139	71	68	51.08		
≥10	53	29	24	54.72		
Insemination method					0.468	0.494
IVF	129	69	60	53.49		
ICSI	63	31	32	49.21		

ICSI = intracytoplasmic sperm injection, IVF = in vitro fertilization, N = no, Y = yes.

Further binary logistic regression analysis showed that age >35 years was an independent factor for clinical pregnancy (*P* < .01; odds ratio [OR] = 1.886 [EXP: 1.479–2.405]). In addition, the type of infertility, number of transplanted embryos, duration of development of transplanted embryos, and thickness of the endometrium were independent factors influencing pregnancy (*P* = .01, OR = 1.370, EXP: 1.088–1.727; *P* < .01, OR = 1.792, EXP: 1.400–2.293; *P* < .01, OR = 2.554, EXP: 1.958, 3.332; *P* = .08, OR = 1.693, EXP: 1.248–2.296, respectively). In these results, the developmental stage of the transplanted embryo was the most significant independent predictor of pregnancy (*P* = .01, OR = 2.554, EXP: 1.958–3.332). These findings are shown in Figure 2A.

Based on the above results, this study analyzed the actual embryo transfer scheme and found that the rates of pregnancy, implantation, and birth after single-blastocyst transfer in fresh embryo cycles were significantly higher than those after other types of transfer. These rates, together with the rate of multiple pregnancies, were also significantly higher after double-blastocyst transfer than after other types of transfers. Similarly, the rates of pregnancy, implantation, and birth after single-blastocyst transfer in FET cycles were significantly higher than those after other types of transfers. In addition, the rates of pregnancy, implantation, and birth after double-blastocyst transfer were significantly higher than those after other types of transfer, as was the rate of multiple pregnancies, The results are shown in Tables 2 and 3.

**Table 2:** Embryo transfer scheme and clinical outcome (fresh IVF cycle).

Fresh IVF cycle	e-SET				e-DET					
	^A^*	^B^*	^C^*	^D^*	^A^*	^E^*	^B^*	^C^*	^F^*	^D^*
N = 192	42	4	22	5	15	100	2	1	0	1
Average age (mean ± SD)	34.43 ± 4.65	38.25 ± 5.38	32.91 ± 3.07	30.80 ± 3.96	33.20 ± 4.74	33.50 ± 4.31	35.00 ± 8.49	31.00	—	32.00
Year of infertility (mean ± SD)	4.02 ± 2.56	5.25 ± 2.87	4.00 ± 2.71	2.60 ± 1.52	4.47 ± 2.07	4.54 ± 3.20	5.00 ± 2.83	3.00	—	2.00
BMI (mean ± SD) (kg/m^2^)	22.75 ± 4.56	22.15 ± 1.87	21.35 ± 3.22	23.94 ± 4.89	21.83 ± 2.24	23.45 ± 2.92	24.85 ± 3.75	20.30	—	22.20
AMH, median (Q_25_, Q_75_) (ng/mL)	1.89 (0.30, 3.44)	0.57 (0.07, 1.02)	2.66 (0.00, 3.32)	4.63 (0.82, 9.79)	1.16 (0.74, 2.99)	1.61 (0.51, 2.52)	1.76 (0.34, —)	2.11	—	1.97
Basic FSH, median (Q_25_, Q_75_) (IU/L)	6.28 (5.16, 7.71)	6.96 (6.89, 16.57)	6.47 (5.94, 7.97)	6.58 (4.52, 7.86)	6.81 (5.73, 9.70)	6.21 (4.96, 7.89)	9.93 (6.81, —)	8.15	—	3.50
Basic E_2_, median (Q_25_, Q_75_) (pmol/L)	43.03 (27.10, 70.77)	57.73 (27.93, 99.14)	40.56 (26.95, 49.95)	30.89 (16.74, 39.55)	30.58 (19.87, 47.36)	37.28 (25.96, 59.28)	15.71 (15.19, —)	79.22	—	48.02
Number of oocytes retrieved, median (Q_25_, Q_75_)	6.50 (2.00, 10.25)	2.00 (1.25, 5.00)	10.00 (7.75, 11.25)	9.00 (5.50, 13.00)	7.00 (3.00, 7.00)	7.00 (4.25, 10.00)	5.50 (4.00, —)	6.00	—	4.00
Clinical pregnancy, n (%)	13 (30.95)	0 (0.00)	12 (54.55)	2 (40.00)	6 (40.00)	64 (64.00)	0 (0.00)	1 (100.00)	—	1 (100.00)
Implantation, n (%)	13 (30.95)	0 (0.00)	12 (54.55)	2 (40.00)	7 (23.33)	86 (43.00)	0 (0.00)	2 (100.00)	—	1 (50.00)
Multiple pregnancies, n (%)	0 (0.00)	0 (0.00)	0 (0.00)	0 (0.00)	1 (16.67)	22 (34.38)	0 (0.00)	1 (100.00)	—	0 (0.00)
Live birth, n (%)	7 (16.67)	0 (0.00)	12 (54.55)	2 (40.00)	4 (26.67)	60 (60.00)	0 (0.00)	2 (100.00)	—	0 (0.00)

A*: 1 good-quality embryo, B*: 0 good-quality embryo, C*: 1 good-quality blastocyst, D*: 0 good-quality blastocyst, E*: 2 good-quality embryos, F*: 2 good-quality blastocysts.

AMH = anti-Mullerian hormone, BMI = body mass index, E_2_ = estradiol, e-DET = elective double embryo transfer, e-SET = elective single-embryo transfer, FSH = follicle-stimulating hormone, IVF = in vitro fertilization, “—” = voiding.

**Table 3:** Embryo transfer scheme and clinical outcome (FET cycle).

FET cycle	e-SET				e-DET							
	^A^*	^B^*	^C^*	^D^*	^A^*	^E^*	^B^*	^C^*	^F^*	^D^*	^G^*	^H^*
N = 1117	48	2	386	115	25	114	4	168	100	113	40	2
Average age (mean ± SD)	36.25 ± 4.34	32.50 ± 2.12	32.96 ± 4.01	34.21 ± 4.59	35.72 ± 5.14	34.91 ± 4.63	34.50 ± 8.89	33.80 ± 4.18	33.26 ± 4.19	34.45 ± 4.38	37.00 ± 4.28	40.00 ± 1.41
Year of infertility (mean ± SD)	3.75 ± 2.85	4.50 ± 2.12	4.30 ± 2.74	4.71 ± 3.52	4.28 ± 3.35	4.14 ± 2.71	3.75 ± 4.27	4.20 ± 2.57	4.23 ± 3.21	5.10 ± 3.86	6.13 ± 4.45	9.50 ± 6.36
Conversion day, endometrial thickness (mean ± SD) (mm)	8.56 ± 1.65	9.00 ± 4.24	8.92 ± 1.69	8.73 ± 1.90	8.54 ± 1.74	8.78 ± 1.79	7.50	8.72 ± 1.61	8.92 ± 1.61	8.86 ± 1.83	7.71 ± 1.57	—
Conversion day, P, median (Q_25_, Q_75_) (ng/mL)	0.29 (0.14, 1.89)	—	0.29 (0.19, 0.43)	0.28 (0.13, 0.40)	1.79 (1.04, —)	0.31 (0.17, 1.18)	—	0.24 (0.18, 0.40)	0.25 (0.17, 0.44)	0.21 (0.14, 0.36)	0.30 (0.16, 0.78)	—
Conversion day, E_2_, median (Q_25_, Q_75_) (pmol/L)	177.95 (18.41, 456.25)	—	222.50 (134.55, 817.30)	256.00 (149.25, 475.75)	161.45 (112.50, —)	255.15 (154.03, 1108.25)	—	304.10 (174.90, 1307.00)	203.10 (152.90, 513.00)	290.05 (170.30, 920.28)	283.20 (198.65, 361.85)	—
Clinical pregnancy, n (%)	9 (18.75)	1 (50.00)	250 (64.77)	44 (38.26)	11 (44.00)	45 (39.47)	0 (0.00)	115 (68.45)	79 (79.00)	62 (54.87)	18 (45.00)	1 (50.00)
Implantation, n (%)	10 (20.83)	1 (50.00)	256 (66.32)	48 (41.74)	11 (22.00)	52 (22.81)	0 (0.00)	156 (46.43)	133 (66.50)	85 (37.61)	25 (31.25)	1 (16.67)
Multiple pregnancies, n (%)	1 (11.11)	0 (0.00)	6 (2.40)	3 (6.82)	0 (0.00)	7 (15.56)	0 (0.00)	40 (34.78)	50 (63.29)	22 (35.48)	6 (33.33)	0 (0.00)
Live birth, n (%)	7 (14.58)	1 (50.00)	148 (50.51)	29 (34.52)	7 (14.29)	39 (19.70)	0 (0.00)	100 (34.97)	102 (53.13)	40 (27.78)	13 (18.57)	1 (16.67)

A*: 1 good-quality embryo, B*: 0 good-quality embryo, C*: 1 good-quality blastocyst, D*: 0 good-quality blastocyst, E*: 2 good-quality embryos, F*: 2 good-quality blastocysts, G*: 1 embryo and 1 blastocyst, H*: 3 embryos/blastocysts.

E_2_ = estradiol, e-DET = elective double embryo transfer, e-SET = elective single-embryo transfer, FET = frozen embryo transfer, P = progesterone, “—” = voiding.

Finally, this study compared and analyzed transfer schemes with the highest pregnancy rates in all transfer cycles, including e-SET (C*) and e-DET (C* + E* + F*). The results showed that there was no statistical difference (*P* = .439) in clinical pregnancy rates between e-SET (C*) and e-DET (C* + E* + F*); however, there was a statistically significant difference (*P* < .01) in multiple pregnancy rates. The results are shown in Figure 2C.

## 4. Discussion

This retrospective study found that the stage of embryo transfer and the age of the women were important influential factors in pregnancy outcomes; the transfer of single high-quality blastocysts can significantly reduce the multiple pregnancy rate while ensuring an ideal pregnancy rate. e-SET (high-quality blastocysts) is worth considering as a more efficient method in all embryo transfer schemes.

Univariate analysis of the factors affecting pregnancy identified the stage of the transplanted embryos and women’s age as the most significant predictors of clinical pregnancy outcomes. In addition, the results of this study showed that the pregnancy outcomes of primary infertility cycles were better than those of secondary infertility cycles, which is unexpected. One possible explanation for this is that there were very different numbers of samples in the 2 groups and the total sample size was small. Notably, age, duration of marriage, and socioeconomic status are reportedly predictors of fertility in women with secondary infertility.^[13]^ Because there were no significant differences in the effects of the total number and days of Gn administration on clinical outcomes in this study, any further statistical analysis of these factors was not performed, but only binary logistic regression analysis on the factors was identified by univariate analysis as having a significant impact on pregnancy outcomes. The results showed that the embryo transfer stage and age of the woman were significant predictors of pregnancy outcome. This is in line with the consensus that age is the primary predictor of clinical pregnancy rates in assisted reproductive cycles.^[14]^ This study also found that the pregnancy rate after the transfer of blastocysts was significantly higher than that after the transfer of embryos, which is in line with common sense. Further embryo culture eliminates embryos of poor quality or those unable to develop into blastocysts^[15]^; however, this cannot be used as a basis for transferring blastocysts in all cycles. The results of the multiple regression analysis indicated that it is necessary to conduct a more specific analysis of the most significant influencing factors. Therefore, this study further analyzed different embryo transfer schemes for fresh and FET cycles and found that pregnancy outcomes and efficacy of transfer were better for 1 high-quality blastocyst than for 1 or 2 cleavage-stage embryos after fresh cycle transfers. Although double-cleavage embryo transfer also achieves a good clinical pregnancy rate, the rate of multiple pregnancies after transplantation is also significantly higher, which is undesirable.^[16]^ Similarly, in FET cycles, pregnancy outcomes and efficiency after the transfer of high-quality blastocysts were better than those after the transfer of 1 or 2 cleavage and blastocyst embryos, especially after the transfer of 2 high-quality blastocysts. Thus, the findings of this study indicated that transplantation of high-quality blastocysts can be considered the first choice for embryo transfer. Notably, regarding fresh cycles, although pregnancy outcomes after high-quality single-blastocyst transfer are relatively good, not all fresh cycles can cultivate high-quality blastocysts. Blastocyst culture eliminates poor-quality embryos. For cycles with few available embryos on day 3, continued blastocyst culture may run the risk of no or very few embryos being available.^[17]^ In addition, continuous culture increases the duration of exposure of embryos to nonoptimal physiological conditions, which may adversely affect subsequent embryonic development.^[18]^ Therefore, informed consent based on the specific characteristics of the cycle is required before further embryo culture or transplantation is performed. According to our findings, plus the fact that e-SET is currently receiving much attention,^[19–21]^ Transplanting 1 high-quality blastocyst is likely the best embryo transfer scheme for patients younger than 35 years with a good prognosis and ideal embryo quality and quantity. The results of Freeman et al^[22]^ also support our conclusions.

One limitation of this study was the small number of fresh embryo transfer cycles. In addition, only classic clinical and laboratory factors potentially influencing pregnancy rates and outcomes (and not other possibly related factors) were analyzed. It is foreseeable that from the perspective of eugenics and technological development, selective high-quality single-embryo transfer in IVF and FET cycles will become a trend in the future. The results of this study can be used as a reference for clinicians and patients when developing embryo transfer schemes.

In conclusion, the results of this study support that the transfer of single high-quality blastocysts can significantly reduce the multiple pregnancy rate while ensuring an ideal pregnancy rate in IVF and FET cycles, which can be used as a reference for planning the first transplantation of patients with good prognoses. With the development of assisted reproductive technology and theory, there will be a more scientific and accurate consensus and norms to guide the selection of transplanted embryos.

## Author contributions

**Conceptualization:** Hao Zhang.

**Writing—original draft:** Hao Zhang.

**Data curation:** Yuanyuan Zhang.

**Software:** Yuanyuan Zhang.

**Resources:** Yaping Cheng.

**Visualization:** Yaping Cheng.

**Investigation:** Hua Yan.

**Project administration:** Xi Zheng.

**Supervision:** Xi Zheng.
